# The Protective Effect of Melittin on Renal Fibrosis in an Animal Model of Unilateral Ureteral Obstruction

**DOI:** 10.3390/molecules21091137

**Published:** 2016-08-27

**Authors:** Hyun-Jin An, Jung-Yeon Kim, Woon-Hae Kim, Sang-Mi Han, Kwan-Kyu Park

**Affiliations:** 1 Department of Pathology, College of Medicine, Catholic University of Daegu, 33, Duryugongwon-ro 17-gil, Nam-gu, Daegu 42472, Korea; ahj119@cu.ac.kr (H.-J.A.); jy1118@cu.ac.kr (J.-Y.K.); kimwoonhae@cu.ac.kr (W.-H.K.); 2 Deparment of Agricultural Biology, National Academy of Agricultural Science, RDA, 300, Nongsaengmyeong-ro, Wansan-gu, Jeonju 54875, Korea; sangmih@korea.kr

**Keywords:** renal fibrosis, melittin, UUO, inflammation

## Abstract

Renal fibrosis is the principal pathological process underlying the progression of chronic kidney disease that leads to end-stage renal disease. Melittin is a major component of bee venom, and it has anti-bacterial, anti-viral, and anti-inflammatory properties in various cell types. Thus, this study examined the therapeutic effects of melittin on the progression of renal fibrosis using the unilateral ureteral obstruction (UUO) model. In addition, the effects of melittin on inflammation and fibrosis in renal fibroblast cells were explored using transforming growth factor-β1 (TGF-β1). Histological observation revealed that UUO induced a considerable increase in the number of infiltrated inflammatory cells. However, melittin treatment markedly reduced these reactions compared with untreated UUO mice. The expression levels of inflammatory cytokines and pro-fibrotic genes were significantly reduced in melittin-treated mice compared with UUO mice. Melittin also effectively inhibited fibrosis-related gene expression in renal fibroblasts NRK-49F cells. These findings suggest that melittin attenuates renal fibrosis and reduces inflammatory responses by the suppression of multiple growth factor-mediated pro-fibrotic genes. In conclusion, melittin may be a useful therapeutic agent for the prevention of fibrosis that characterizes the progression of chronic kidney disease.

## 1. Introduction

Renal fibrosis is the principal pathological process underlying the progression of chronic kidney disease that leads to end-stage renal disease [1]. Many studies have revealed that renal fibrosis progression is mediated by multiple mechanisms [2]. Renal fibrosis is the final stage of renal injury that results from various etiologies, such as diabetic nephropathy, hypertensive glomerulosclerosis, and chronic renal allograft nephropathy, among others [3,4]. These processes are characterized by the infiltration of inflammatory cells, interstitial accumulation of fibroblasts, proliferation of myofibroblasts, deposition of the extracellular matrix (ECM), and loss of renal tubule epithelial cells, which collectively lead to end-stage renal failure [5]. 

Renal fibrosis is initially caused by inflammation of the tubulointerstitial compartments, which plays a major role in the progression of renal disease [6]. Aggravated inflammation eventually turns into interstitial fibrosis, which leads to the complete destruction of renal parenchyma and the disruption of renal function. Multiple factors are implicated in renal inflammation, including cytokines, chemokines, and growth factors [7]. Many different types of cytokines and growth factors are involved; particularly, tumor necrosis factor-α (TNF-α) and transforming growth factor-β1 (TGF-β1) play key roles as mediators of renal interstitial fibrosis by regulating ECM production [8]. 

TNF-α is a multifunctional cytokine that promotes inflammation, and this inflammatory reaction is often involved in the initiation and progression of fibrosis [9,10]. Of the numerous growth factors involved in the pathophysiology of renal fibrosis, TGF-β1 is considered a crucial mediator in the initiation and progression of interstitial fibrosis [11]. TGF-β1 mediates progressive renal fibrosis by stimulating ECM production, such as collagen type I and fibronectin, while inhibiting the degradation of ECM [12]. In addition, TGF-β1 induces the activation of fibroblasts to undergo a phenotypic transition to myofibroblasts, which are the effectors of the fibrotic state and are characterized by their association with contractile proteins, such as α-smooth muscle actin (α-SMA) and non-muscle myosin [13]. Myofibroblasts are fully differentiated fibroblasts, which are α-SMA-positive cells and the main source for the ECM [14]. Therefore, blocking the fibrotic response by inhibiting gene expression or the production of related proteins or receptors may prevent or reverse this damage [3]. In this study, a therapeutic intervention that blocks the activation of multiple cytokines and growth factors (such as TGF-β1 and TNF-α) might improve anti-fibrotic effects and help slow the progression of renal fibrosis. 

Bee venom is a natural toxin produced by honeybees (*Apis mellifera*), and has been widely used as a traditional medicine to treat various diseases [15,16]. Bee venom contains a variety of peptides, including melittin, apamin, adolapin, and mast cell degranulating peptide, along with enzymes, biological amines, and non-peptide components [17]. Melittin is a major component of bee venom, comprising 50% of its dry weight [18]. It is a small linear peptide composed of 26 amino acid residues. Many studies have examined the biological and pharmacological activities of melittin; it has anti-bacterial, anti-viral, and anti-inflammatory properties in various cell types [19]. In addition, it has anti-rheumatoid arthritis effects, pain-relief effects, anti-cancer cell proliferation, and aids immune modulatory activity [15,20,21,22]. However, the precise mechanism of melittin in ameliorating the renal fibrosis is not fully understood. 

Therefore, this study investigated the anti-fibrotic effects of melittin on the expression of inflammatory cytokines and on the activation of growth factors related to the development of progressive renal fibrosis in an animal model of unilateral ureteral obstruction (UUO). 

## 2. Results

### 2.1. Melittin Attenuates Renal Interstitial Injury and Fibrosis in UUO Kidneys

**Figure 1:**
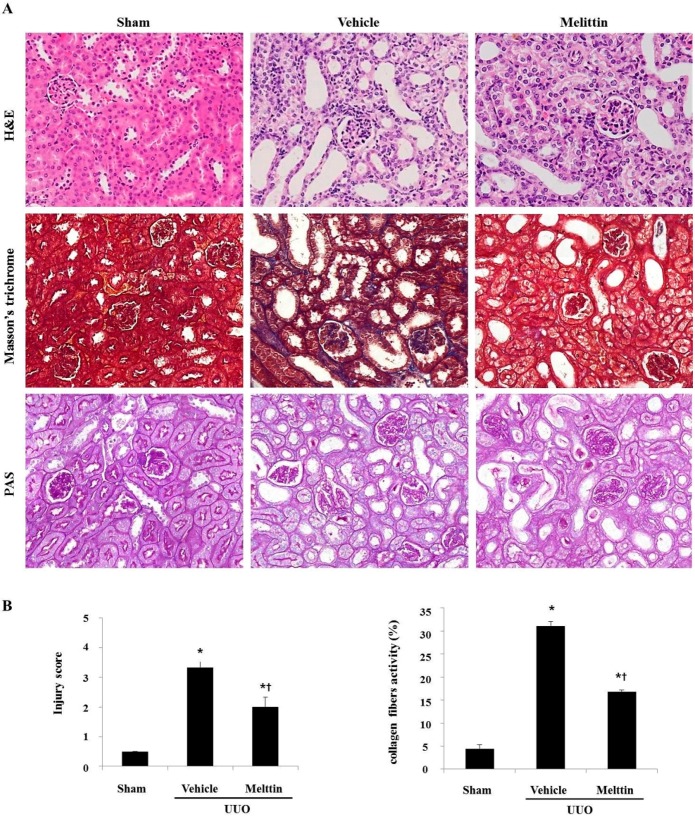
The effects of melittin on histological alterations in unilateral urethral obstruction (UUO) kidneys. Histopathological alterations in the hematoxylin and eosin (H&E) stained, the Masson’s trichrome-stained, and the periodic acid–Schiff (PAS) stained slides (**A**). The kidney sections are stained with Masson’s trichrome, which accentuates interstitial fibrosis by staining the collagen blue. (**B**) The semi-quantitative analysis of the interstitial injury score and relative fibrogenic area of the obstructed kidney in each group. These are representative images from each study group. Magnification 400×. The results are expressed as means ± SE of three independent determinations. * *p* < 0.05 vs. Sham group. † *p* < 0.05 vs. Vehicle group.

Glomerular sclerosis and vascular sclerosis are two major pathogenetic changes in the UUO model [23]. To investigate the components responsible for the effects of melittin, the mouse model of UUO was examined. We next performed histopathological analysis using hematoxylin and eosin (H&E) stains, Masson’s trichrome stains, and periodic acid–Schiff (PAS) stains in UUO mice. The Sham group showed normal kidney histology. In UUO kidneys, the glomerulus was damaged, and segmental sclerosis of the glomerular tuft was apparent. In addition, UUO kidneys showed signs of severe tubulointerstitial injuries, including tubular dilatation, atrophy, and interstitial fibrosis. However, melittin treatment significantly reduced these changes compared with the Vehicle group (Figure 1A). UUO also induced collagen in the kidneys, as exhibited by Masson’s trichrome staining. Melittin administration markedly reduced the area of tubulointerstitial fibrosis in the kidney in the Vehicle group. These findings were confirmed by quantification assessment of the tubulointerstitial damage observed in the H&E stained sections and Masson’s trichrome stained sections of the renal tissue (Figure 1B). Taken together, these results indicate that melittin treatment of renal fibrosis in mice improved renal function and delayed renal damage and fibrosis.

### 2.2. Melittin Suppresses Pro-Inflammatory Cytokines in Kidneys after UUO

**Figure 2:**
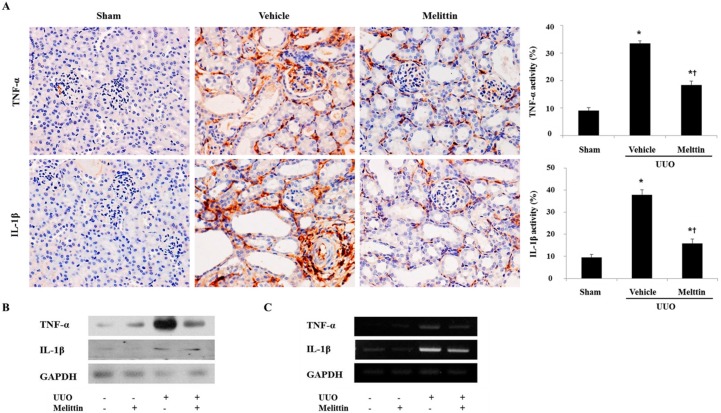
Melittin attenuates the expression of pro-inflammatory cytokine in obstructed kidneys. (**A**) Immunohistochemical staining shows that melittin inhibits the expressions of tumor necrosis factor (TNF)-α and interleukin (IL)-1β in kidneys at 7 days after unilateral ureteral obstruction (UUO) surgery. Immunohistochemical staining was used to evaluate the extent of pro-inflammatory cytokines, which was subsequently quantified. (**B**, **C**) Western blot analysis and Reverse transcription-polymerase chain reaction (RT-PCR) results show that melittin suppresses the protein and mRNA expressions of TNF-α and IL-1β in UUO kidneys. Glyceraldehyde-3-phosphate-dehydrogenase (GAPDH) levels were analyzed as an internal control. −: not treated, +: treated. These are representative images from each study group. Magnification: 400×. The results are expressed as means ± SE of three independent determinations. * *p* < 0.05 vs. Sham group. † *p* < 0.05 vs. Vehicle group.

To investigate the effects of melittin on the inflammatory changes caused by UUO, melittin was administrated to the mouse model of UUO. As shown in Figure 2A, immunohistochemistry revealed a marked increase in the accumulation of TNF-α and interleukin (IL)-1β in the kidneys of the Vehicle group. However, melittin treatment markedly decreased their expressions in UUO kidneys. Further, melittin-treated kidneys had significantly reduced expressions of TNF-α and IL-1β compared with UUO kidneys (Figure 2B,C). There were no obvious expression changes in the kidneys of both the Sham group and the melittin alone-treated group. Thus, these observations demonstrated that melittin effectively inhibited the expressions of pro-inflammatory cytokines in the mouse model of renal fibrosis.

### 2.3. Melittin Inhibits Fibrotic Gene Expression in the Animal Model of Renal Fibrosis

**Figure 3:**
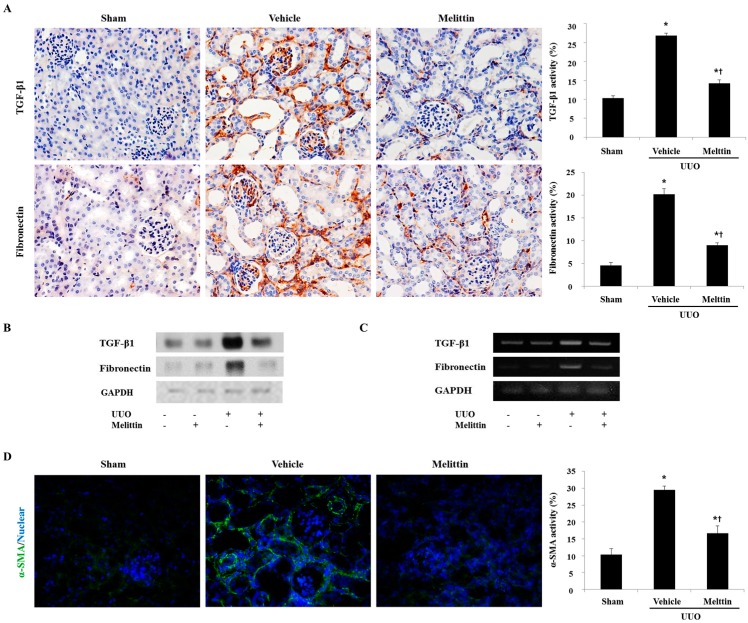
Melittin inhibits the expression of the fibrotic gene in obstructed kidneys. (**A**) Immunohistochemical staining shows that melittin inhibits the expression of transforming growth factor-β1 (TGF-β1) and fibronectin in the kidneys after UUO surgery. (**B**, **C**) Western blot analysis and RT-PCR results shows that melittin suppresses the protein and mRNA expression of TGF-β1 and fibronectin in UUO kidneys. GAPDH levels were analyzed as an internal control. −: not treated, +: treated. (**D**) Immunofluorescence staining shows that melittin treatment reduces α-smooth muscle actin (α-SMA)-positive cells in the kidneys at seven days after UUO surgery. The visible green color indicates α-SMA. These are representative images from each study group. Magnification: 400×. The results are expressed as means ± SE of three independent determinations. * *p* < 0.05 vs. Sham group. † *p* < 0.05 vs. Vehicle group.

This study next examined the effect of melittin on fibrosis-related gene expression. Fibronectin is the first ECM protein that is deposited in fibrogenesis [24]. The number of cells positive for TGF-β1 and fibronectin were increased in the Vehicle group, but decreased after melittin treatment. In particular, TGF-β1 and fibronectin were strongly positive in the epithelial cells of the dilated tubules in UUO kidneys (Figure 3A). As shown in Figure 3B,C, the expressions of TGF-β1 and fibronectin increased in the UUO kidney; however, this increase was abolished by melittin treatment in UUO mice. These results suggest that melittin treatment has renoprotective properties and is able to prevent the development of fibrotic lesions in UUO kidneys. The generation of myofibroblasts is considered a key process in tubulointerstitial fibrosis, which accounts for the accumulation of the ECM under diseased conditions [25]. Thus, myofibroblasts can be used as prognostic indicators of renal disease progression [14]. To investigate the ability of melittin to suppress myofibroblast activation, this study examined the expression of α-SMA—a representative marker of activated myofibroblasts—by immunofluorescent staining (Figure 3D). The Vehicle group displayed an increased number of α-SMA-positive cells, whereas this population of cells was significantly reduced by melittin treatment. Therefore, these data suggest that melittin has a potent capability to inhibit the activation of renal fibroblasts in vivo. 

### 2.4. Anti-Fibrotic Effects of Melittin in Renal Fibroblast Cell

**Figure 4:**
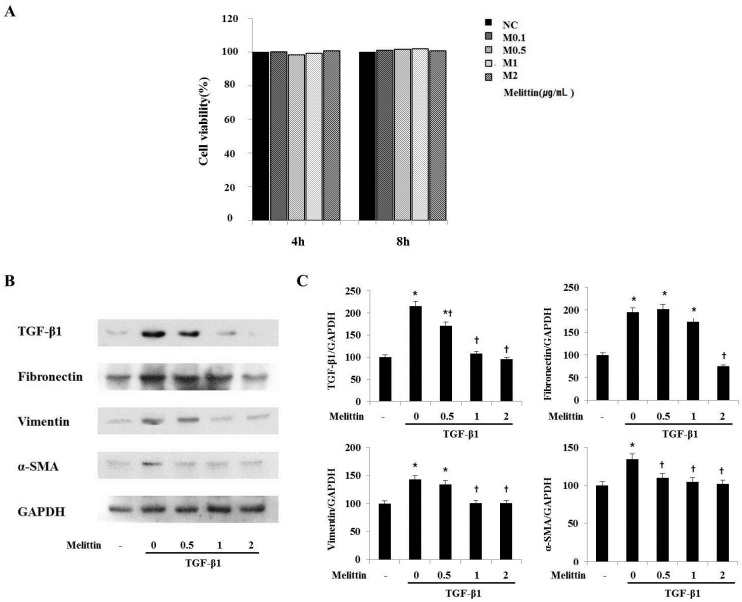
Melittin treatment suppresses fibrosis-related gene expression in renal fibroblasts. (**A**) The cytotoxic effects of melittin on renal fibroblast NRK-49F cells. The cytotoxicity of melittin was determined by an MTT assay in NRK-49F cells. NRK-49F cells were treated with 0.1, 0.5, 1, and 2 μg/mL of melittin for 4 and 8 h. (**B**) A Western blot analysis showed that melittin treatment effectively suppressed the expressions of TGF-β1, fibronectin, vimentin, and α-SMA in TGF-β1-induced NRK-49F cells fibrosis. (**C**) The expression levels of the indicated proteins were quantified by densitometry and normalized with GAPDH. −: not treated. The results are expressed as mean ± S.E. of three independent determinations. *p < 0.05 vs. Normal, control. † *p* < 0.05 vs. Melittin concentration = 0.

Earlier work demonstrated that TGF-β1 is the most important factor in fibrogenesis for inducing and propagating various cytokines [6]. The effects of melittin on inflammation and fibrosis in fibroblast cells were explored using TGF-β1. Based on the results of melittin in the UUO model, this study investigated the effects of melittin on TGF-β1-induced inflammation and fibrosis in NRK-49F cells. An MTT (3-(4,5-Dimethylthiazol-2-yl)-2,5-Diphenyltetrazolium Bromide) assay was carried out to determine the cytotoxicity of melittin in NRK-49F cells. Thus, NRK-49F cells were treated with melittin at 0.1, 0.5, 1, and 2 μg/mL for 4 and 8 h. After treatment with melittin, it did not affect the cell viability of NRK-49F cells. In addition, different concentrations of melittin treatment did not change the viability of NRK-49F cells. Thus, the effects of melittin on NRK-49F cells were minimal at 8 h. As shown in Figure 4B, in TGF-β1-injury, NRK-49F cells increased the protein level expressions of TGF-β1, fibronectin, vimentin, and α-SMA. These results were decreased by melittin treatment (0.5, 1, and 2 μg/mL). More specifically, 2 μg/mL of melittin resulted in a significant decrease in the expression of TGF-β1, fibronectin, vimentin, and α-SMA. Therefore, these observations demonstrated that melittin effectively inhibited fibrosis-related gene expression in renal fibroblasts. These data support the in vivo experiment that revealed melittin as a potentially effective inhibitor of renal fibrosis.

## 3. Discussion

Animal models of chronic kidney disease provide the opportunity to investigate disease-specific mechanisms and molecular pathogenesis and to assess potential novel therapies [26]. One of the most frequent experimental models for the investigation of interstitial fibrosis are mouse UUO models [3]. The UUO model is useful for the examination of mechanisms of interstitial fibrosis and reducing the glomerular filtration rate. Blood flow induces interstitial inflammation and eventually leads interstitial fibrosis [27]. Complete UUO initiates a rapid sequence of events in the obstructed kidney, leading to reduced renal blood flow and a reduced glomerular filtration rate within 24 h [28]. As previously described, UUO-operated kidneys show interstitial inflammation after 3 days; tubular dilation, tubular atrophy, and fibrosis after 7 days; and end-stage renal failure approximately 2 weeks after UUO operation [27]. The UUO model was chosen for this study because the procedure has similar characteristics to human obstructive nephropathy [3]. 

Recent reports have shown that obstruction-mediated renal injuries are involved in the mechanisms of inflammatory cytokines, chemokines, and fibrosis-related gene expressions [29]. Further, a recent study indicated that melittin prevents the DDC diet-induced expression levels of inflammatory cytokines and fibrosis-related genes. This study demonstrated the protective effects of melittin on DDC-induced biliary fibrosis in vivo [30]. On the basis of this information, our current study investigated the question of whether melittin could affect inflammation and fibrosis reaction after renal injury. To investigate the effects of melittin on inflammation and fibrosis in UUO kidneys, melittin was administrated to mouse UUO models.

The pathophysiology of renal fibrosis can be divided into four phases: (1) cellular activation and injury (priming); (2) fibrogenic signaling (activation); (3) fibrogenesis (execution); and (4) destruction (progression) [1]. Renal interstitial fibrosis—a common hallmark of progressive chronic renal disease—is characterized by inflammatory cell infiltration, myofibroblast proliferation, and accumulation of ECM [31]. Infiltrated inflammatory cells not only generate numerous chemokines, but also release pro-fibrotic cytokines and growth factors (e.g., TGF-β1), which then act on renal tubular cells and resident fibroblasts to promote renal fibrosis. TGF-β1, which recruits and activates fibroblasts, is well known as a potent pro-fibrogenic cytokine that mediates fibrosis in multiple organs, including the lungs, liver, and kidneys [32]. Thus, TGF-β1 is the most potent mediator and convergent pathway in renal fibrosis [33]. In the present study, UUO kidneys showed an increased expression of TGF-β1 and an increased production of fibronectin, a major ECM protein, compared with the Sham group. However, this increase was abolished by melittin treatment. 

Mechanical stress, cytokines, and various other factors induce fibroblasts to acquire a myofibroblast phenotype [34]. Since myofibroblasts play a key role in fibrosis, the expression of α-SMA (a marker of myofibroblast differentiation) was examined by immunofluorescence in UUO mice treated with melittin. UUO-induced renal myofibroblast activation was significantly suppressed in melittin-treated mice. Further, the results of our in vitro studies support the hypothesis that melittin plays an essential role in the suppression of renal fibrosis. 

In the present study, we explored the potential of melittin as a therapeutic candidate using the UUO-induced interstitial fibrosis model. The expression levels of inflammatory cytokines (e.g., TNF-α and IL-1β) and pro-fibrotic genes (e.g., TGF-β1 and fibronectin) were significantly reduced in melittin-treated mice compared to UUO mice. We demonstrated that melittin treatment suppressed renal inflammation and ECM deposition after UUO. Collectively, melittin prevented the development and progression of renal interstitial fibrosis in UUO models. The beneficial effects of melittin may be mediated primarily or in part by its ability to block the activation of TGF-β and other inflammatory cytokines and the consequential degradation of the ECM-accumulation pathway. Melittin also effectively inhibited fibrosis-related gene expression in renal fibroblasts NRK-49F cells. Based on these results, melittin could be an effective alternative treatment for renal fibrogenesis. Further investigations are needed to evaluate whether the anti-fibrotic action of melittin is also associated with the inhibition of fibrosis-related receptors in the kidney. In conclusion, melittin may be a useful therapeutic agent for the prevention of fibrosis that characterizes progression of chronic kidney disease.

## 4. Materials and Methods 

### 4.1. Animal Model

The animal models were male BALB/c mice (20–25 g) that were individually housed in polycarbonate cages and maintained under a constant temperature (22 ± 2 °C) and humidity (55%). The mice had free access to food and water and were subjected to an artificial light–dark cycle of 12:12 h. After 1 week of acclimatization, the BALB/c mice were randomly divided into four groups: the first group was anesthetized and underwent a similar surgical procedure of UUO but was not subjected to ureteral ligation (Sham); the second group was subjected to a Sham group with a melittin (Sigma-Aldrich, St. Louis, Mo, USA) treatment; the third group underwent a vehicle treatment for UUO (Vehicle); and the fourth group consisted of UUO mice treated with melittin (Melittin) (*n* = 6 in each group). In the UUO operation, the anesthetized animal‘’s abdominal cavity was exposed by a midline incision, and the left ureter was isolated and ligated with 5-0 silk suture thread at two different sites: distal and proximal. An intraperitoneal injection of melittin at a concentration of 0.01 mg/kg was given immediately after ureteral ligation. Then, melittin was given with an intraperitoneal injection 2 days after the UUO operation. The kidneys were collected for mRNA and protein analyses, including a histologic examination, on day 7 post-UUO surgery. All surgical and experimental procedures used in the current study were approved by the institutional animal care and use committee at the Daegu Catholic University Medical Center. 

### 4.2. Histological Analysis

All kidney tissue specimens were fixed in 10% formalin for at least 24 h at room temperature. After fixation, sections perpendicular to the anterior–posterior axis of the kidney were dehydrated in graded ethanol, cleared in xylene, and embedded in paraffin. Thin sections (4 μm) were mounted on glass slides, dewaxed, rehydrated to distilled water, and stained with H&E and PAS. As part of the histological evaluation, all slides were examined under a light microscope. Histological changes were mainly evaluated by a quantitative measurement of the tubular injury (magnification: ×200). The histopathologically altered H&E stained slides were scored by a quantitative percentage of the damaged area, as follows: 0, 0%–5%; 1, 5%–10%; 2, 11%–25%; 3, 26%–45%; 4, 46%–75%; and 5, >76%. The three fields analyzed in each section were selected at random. 

For the Masson’s trichrome stain, the tissue sections were deparaffinized and refixed in Bouin’s solution for 1 h at 56–60 °C. After being stained with Weigert’s hematoxylin and a Biebrich scarlet-acid fuchsin, the sections were treated in phosphomolybdic–phosphotungstic acid and finally stained with aniline blue. To evaluate tubulointerstitial collagen deposition, three randomly selected fields in each section stained with Masson’s trichrome were analyzed at ×400 magnification. The area stained in light blue in the interstitium was semiquantitatively calculated using i-Solution Lite V.9.1 Image Analysis Software (IMTechnology, Vancouver, BC, Canada).

### 4.3. Immunohistochemical Staining

Paraffin-embedded tissue sections at 4 μm thickness were deparaffinized with xylene, dehydrated in gradually decreasing concentrations of ethanol, and then treated with 3% hydrogen peroxidase in methanol for 10 min to block endogenous peroxidase activity. The tissue sections were immersed in a 10 mM sodium citrate buffer (pH 6.0) for 5 min at 95 °C. The last step was repeated using a fresh 10 mM sodium citrate solution (pH 6.0). The sections were allowed to remain in the same solution while cooling for 20 min, and they were then rinsed in phosphate-buffered saline (PBS). After, the sections were incubated with a primary antibody (1:100 dilution) for 1 h at 37 °C. The primary antibodies were follows: anti-TNF-α and anti-fibronectin (Abcam, Cambridge, MA, USA), anti-IL-1β (Santa Cruz Biotechnology, Dallas, TX, USA), and anti-TGF-β1 (R&D Systems, Minneapolis, MN, USA). The signal was visualized using an Envision System (DAKO, Carpinteria, CA, USA) for 30 min at 37 °C. 3,3′-diaminobenzidine tetrahydrochloride (DAB) was used as the coloring reagent, and hematoxylin was used as the counter-stain.

### 4.4. Immunofluorescent Staining

The paraffin-embedded tissue sections were deparaffinized with xylene and dehydrated in gradually decreasing concentrations of ethanol. The tissue sections were then placed in a blocking serum (5% bovine serum albumin in PBS) at room temperature for 1 h. A primary antibody (1:500 dilution) was incubated at room temperature for 2 h, and a secondary antibody incubation (1:200 dilution) was performed at room temperature for 2 h. The antibodies included α-SMA (Abcam), and a goat anti-mouse secondary antibody conjugated with fluorescein isothiocyanate (FITC) (Invitrogen, Carlsbad, CA, USA). Sections were then counterstained with Hoechst 33342. The slides were mounted using a VECTASHIELD Mounting Medium (VECTOR Laboratories, Burlingame, CA, USA), and the specimens were examined and photographed using a fluorescence microscope (Nikon, Tokyo, Japan). 

### 4.5. Western Blot Analysis

The kidney tissues were homogenized in a lysis buffer (50 mM Tris pH 8.0, 150 mM NaCl, 5 mM EDTA, 0.5% NP-40, 100 mM phenylmetylsulfonyl fluoride (PMSF), 1 M Dithiothreitol (DTT), 10 mg/mL leupeptin and aprotinin; Sigma-Aldrich, St Louis, MO, USA). After incubation for 30 min on ice, the samples were centrifuged at 12,000 rpm for 20 min at 4 °C. Then, the supernatant was collected and the residual protein concentration was measured by the Bradford protein assay (Bio-Rad Laboratories, Hercules, CA, USA). The total protein (10–50 μg) was separated on 8%–12% sodium dodecyl sulfate polyacrylamide gels and transferred to a nitrocellulose membrane (Millipore Corporation, Bedford, MA, USA). The membranes were blocked in 5% skim milk in TBS-T (10 mM Tris, 150 mM NaCl, and 0.1% Tween-20) for 2 h at room temperature. Then, the membrane was probed with a primary antibody overnight at 4 °C, and a horseradish peroxidase-conjugated secondary antibody was used for detection. The signals were detected using an enhanced chemiluminescence detection system (Amersham, Piscataway, NJ, USA). The primary antibodies used in this study were as follows: anti-TNF-α, anti-fibronectin, anti-α-SMA (Abcam), anti-TGF-β1 (R&D Systems), anti-IL-1β, anti-collagen type I, and anti-glyceraldehyde-3-phosphate-dehydrogenase (GAPDH) (Santa Cruz Biotechnology). All primary antibodies were diluted at 1:1000. The signal intensity was quantified by an image analyzer (Las 3000, Fujifilm, Tokyo, Japan).

### 4.6. Reverse Transcription-Polymerase Chain Reaction (RT-PCR)

The total RNA was extracted from the frozen kidney with TRIzol Reagent (Gibco, Grand Island, NY, USA) according to the manufacturer’s recommendations. Both the purity and quantity of the RNA preparation were measured at optical densities of 260 nm and 280 nm. First, the strand cDNA was synthesized with an oligo-(dT) primer and M-MLV reverse transcriptase (Promega, Madison, WI, USA). Aliquot cDNA was used for the PCR using primer sets specific to mouse TNF-α, IL-1β, TGF-β1, fibronectin, and GAPDH. The primer sequences were as follows: TNF-α forward primer, 5′-AGT GGT GCC AGC CGA TGG GTT GT-3′; TNF-α backward primer, 5′-GCT GAG TTG GTC CCC CTT CTC CAG-3′; IL-1β forward primer, 5′-CAT GAG CAC CTT CTT TTC CT-3′; IL-1β backward primer, 5′-TGT ACC AGT TGG GGA ACT CT-3′; TGF-β1 forward primer, 5′-CCT GCT GCT TTC TCC CTC AAC C-3′; TGF-β1 backward primer, 5′-CTG GCA CTG CTT CCC GAA TGT C-3′; fibronectin forward primer, 5′-TGT GAC AAC TGC CGT AGA CC-3′; fibronectin backward primer, 5′-GAC CAA CTG TCA CCA TTG AGG-3′; GAPDH forward primer, 5′-GTG GAC ATT GTT GCC ATC AAC G-3′; and GAPDH backward primer, 5′-GAG GGA GTT GTC ATA TTT CTC G-3′. The PCR products were visualized by 1.5% agarose gel electrophoresis with ethidium bromide staining.

### 4.7. Cell Culture and Viability Assay

Normal rat kidney fibroblast cells (NRK-49F) were obtained from the American Type Culture Collection (Rockville, MD, USA) and cultured in Dulbecco’s Modified Eagle Medium (Gibco BRL, Gaithersburg, MD, USA) containing 10% fetal bovine serum (Gibco BRL), 100 μg/mL penicillin, and 100 μg/mL of streptomycin. The cells were incubated in a 95% air- and 5% CO_2_-humidified atmosphere at 37 °C. The NRK-49F cells were seeded in a 96-well plate at 5 × 10^3^ cells/well and allowed to attach for 24 h. Then, the medium was replaced with serum-free media. After 24 h of serum starvation, the cells were treated with serum-free media containing Mel (0.5, 1, and 2 μg/mL) for 30 min, followed by a treatment with 5 ng/mL of TGF-β1 for 8 h. The cells were then washed with PBS, and the MTT solution (3-(4,5-dimethylthiazol-2-yl)-2,5-diphenyltetrazolium bromide/PBS) was added to each well. The plates were incubated for 4 h at 37 °C. Finally, the MTT-containing medium was removed by aspiration, and 100 μL of dimethylsulfoxide solution was added to each well. The absorbance value was measured at 540 nm using a microplate reader (BMG labtechnologies, Mornington, Germany).

### 4.8. Statistical Analysis

The data are presented as means ± SE. A Student’s *t*-test was used to assess the significance of independent experiments. The criterion *p* < 0.05 was used to determine statistical significance.
